# A transcriptomics-based RNAi screen for regulators of meiosis and early stages of oocyte development in *Drosophila melanogaster*

**DOI:** 10.1093/g3journal/jkae028

**Published:** 2024-02-09

**Authors:** Stacie E Hughes, Andrew Price, Salam Briggs, Cynthia Staber, Morgan James, Madelyn Anderson, R Scott Hawley

**Affiliations:** Stowers Institute for Medical Research, Kansas City, MO 64110, USA; Stowers Institute for Medical Research, Kansas City, MO 64110, USA; Stowers Institute for Medical Research, Kansas City, MO 64110, USA; Stowers Institute for Medical Research, Kansas City, MO 64110, USA; Stowers Institute for Medical Research, Kansas City, MO 64110, USA; Stowers Institute for Medical Research, Kansas City, MO 64110, USA; Stowers Institute for Medical Research, Kansas City, MO 64110, USA; Department of Molecular and Integrative Physiology, University of Kansas Medical Center, Kansas City, KS 66160, USA

**Keywords:** *Drosophila melanogaster*, meiosis, synaptonemal complex, oocyte specification, development

## Abstract

A properly regulated series of developmental and meiotic events must occur to ensure the successful production of gametes. In *Drosophila melanogaster* ovaries, these early developmental and meiotic events include the production of the 16-cell cyst, meiotic entry, synaptonemal complex (SC) formation, recombination, and oocyte specification. In order to identify additional genes involved in early oocyte development and meiosis, we reanalyzed 3 published single-cell RNA-seq datasets from *Drosophila* ovaries, using *vasa* (germline) together with *c(3)G*, *cona*, and *corolla* (SC) as markers. Our analysis generated a list of 2,743 co-expressed genes. Many known SC-related and early oocyte development genes fell within the top 500 genes on this list, as ranked by the abundance and specificity of each gene's expression across individual analyses. We tested 526 available RNAi lines containing shRNA constructs in germline-compatible vectors representing 331 of the top 500 genes. We assessed targeted ovaries for SC formation and maintenance, oocyte specification, cyst development, and double-strand break dynamics. Six uncharacterized genes exhibited early developmental defects. SC and developmental defects were observed for additional genes not well characterized in the early ovary. Interestingly, in some lines with developmental delays, meiotic events could still be completed once oocyte specificity occurred indicating plasticity in meiotic timing. These data indicate that a transcriptomics approach can be used to identify genes involved in functions in a specific cell type in the Drosophila ovary.

## Introduction

The production of haploid gametes is crucial for the success of a species. In the *Drosophila melanogaster* ovary, multiple developmental and meiotic events must occur during early prophase I to have a successful outcome for the egg (Spradling 1993; McLaughlin and Bratu 2015; Hughes *et al*. 2018; Cabrita and Martinho 2023). Considerable time (decades) and effort have been spent on genetic screens in Drosophila to identify mutations that affect early developmental and meiotic processes (see Spradling 1993; Hudson and Cooley 2014; Hughes *et al*. 2018).

The first meiotic mutant to be identified was *c(3)G* (Gowen and Gowen 1922). Almost 80 years later, Page and Hawley (2001) demonstrated that the *c(3)G* gene encodes the major transverse filament protein of the synaptonemal complex (SC). The SC is a large tripartite protein structure that aligns the homologous chromosomes in most sexual organisms. The Drosophila SC has 2 lateral elements that interact with the chromosomes, which are connected by C(3)G within the central region. Cona and Corolla are located within the central region and stabilize the SC (Page *et al*. 2008; Collins *et al*. 2014).

Numerous subsequent screens have identified meiotic genes encoding SC components, meiotic cohesin complexes, and proteins of the recombination machinery in Drosophila females (reviewed in Hughes *et al*. 2018). However, based on a comparison with the number of SC and recombination genes identified in many other organisms [reviewed in Gao and Colaiacovo (2018); Lascarez-Lagunas *et al*. (2020); Arter and Keeney (2023)], we hypothesize that there may be numerous additional meiotic genes yet to be identified in Drosophila. Additional mutagen-based genetic screens may not be the most efficient means to find them. For example, a large mutagen-based screen failed to identify new alleles of several known meiotic genes, including *c(3)G* (Page *et al*. 2007). Many meiotic genes evolve rapidly at the sequence level, which has made homolog searches challenging for identifying the components of the SC and parts of the recombination machinery (Hemmer and Blumenstiel 2016; Kursel *et al*. 2021). In addition, full or partial redundancy of SC and recombination proteins has been observed for meiotic genes (Lake *et al*. 2019; Hurlock *et al*. 2020).

Numerous screens have been conducted to identify genes regulating early ovarian development [reviewed in Spradling (1993); Hudson and Cooley (2014)]. As some of the genes required for early ovarian development may also play roles in somatic tissues, where mutations result in lethality, there may be additional developmental genes that have been missed using mutagen-based screening strategies that require survival to adulthood. Additionally, if proteins function at multiple points in ovarian development and meiosis, null mutations would likely only reveal the earliest function of the protein. Analysis of hypomorphic conditions could provide a means of discovery of additional, later functions in either development or meiosis. For example, strong loss-of-function alleles of *mei-P26* cause defects in germ cell differentiation and proliferation. However, the first *mei-P26* allele identified was a weak loss-of-function mutation that caused defective recombination. The hypomorphic mutation revealed the later role of the Mei-P26 protein (Page *et al*. 2000; Neumuller *et al*. 2008).

Toward this goal of discovering additional genes with roles in cells at the early meiotic/pachytene stage in the Drosophila ovary, we have devised an RNAi screen based on data obtained from single-cell transcriptomic analysis. Specifically, we reanalyzed 3 recently published single-cell (sc) RNA-seq datasets from *Drosophila melanogaster* ovaries using *vasa* (germline) and *c(3)G*, *cona*, and *corolla* (SC) as markers for cells in early meiotic prophase I (Rust *et al*. 2020; Slaidina *et al*. 2020, 2021). Our analysis generated a list of 2,739 genes co-expressed with these marker genes. Reassuringly, 13 of the 14 known genes of the SC central element, lateral element, and axial element, as well as many genes known to function in oocyte development, were included within the top 500 genes, as ranked by the abundance and cell type specificity of each gene across individual analyses, indicating that the analysis correctly identified genes expressed in early meiotic cells. As the top 500 genes on the list contained many known oocyte development and meiosis genes, one can speculate that other genes on this list, many of which have uncharacterized or less characterized roles in the ovary, may also have functions in these processes.

To characterize the roles of the genes that we identified in this analysis in early developmental and meiotic processes, we have used RNAi lines containing shRNA constructs in germline compatible vectors to examine SC formation, SC maintenance, and double-strand break (DSB) dynamics in oocytes bearing such knockdown constructs. Specifically, we used publicly available constructs representing approximately two-thirds of the top 500 genes. As the regulation of meiotic events is dependent on the successful completion of steps of oocyte development, lines causing defects in either SC or DSB processes were further examined for Orb localization to assess cyst development and oocyte specification. Oocyte development defects were identified for 6 uncharacterized genes. Meiotic defects were observed for genes not previously well characterized for these processes.

These results support the idea that our analysis has substantially enriched for genes required in early meiotic cells and indicates that single cell transcriptomics can be used as a method for identifying new genes in biological processes occurring in a defined cell population.

## Materials and methods

### Bioinformatic analysis

Three published scRNA-seq datasets with multiple replicates (9 total) were retrieved as fastq files from the NCBI Short Read Archive using fastq-dump (Rust *et al*. 2020; Slaidina *et al*. 2020, 2021) (Supplementary Table 1). The published datasets were labeled Rust-adult, Slaidina-adult, and Slaidina-larval. SRR9161641 and SRR9161642 were pre-processed with CellRanger as described in Slaidina *et al*. (2021) (https://genome.cshlp.org/content/31/10/1938.full). The rest of the raw fastqs were aligned to the dm6 assembly from UCSC using Ensembl 98 annotations with CellRanger count (3.0.0). Downstream analysis was completed in R (4.1.0), primarily using the Seurat package (4.0.5). For quality control, cells were filtered for downstream analysis based on cutoffs derived from each individual replicate's number of genes expressed and mitochondrial expression distributions (Supplementary Table 1). Each replicate was prepped using the sctransform function in Seurat and then integrated together using Seurat's integration pipeline. Four total separate integration analyses were completed. Three analyses, one for each of the 3 datasets, looked at the replicates within each data set integrated together. The final analysis includes all 9 replicates across the 3 datasets integrated together. The Seurat parameters for these individual analyses can be found in Supplementary Table 2.

In each of the 4 analyses, Seurat RunUMAP was run to reduce the dimensionality of the data into a 2-dimensional space. Clusters were then identified using Seurat FindClusters. Germline clusters were labeled using the expression of established germline and SC-specific marker genes [*vas*, *cona*, *corolla*, and *c(3)G*]. Differentially expressed positive genes for the germline clusters were identified using Seurat FindMarkers and were filtered at a Bonferroni-corrected adjusted *P*-value of <0.05. The genes were then ranked in each analysis based on having the lowest adjusted *P*-value.

To find strong, consistent candidate genes present across all datasets, the gene lists were intersected, and only significant germline genes found in all 4 of the individual analyses were kept. These genes were then ordered by calculating the mean rank of the gene in the individual analyses and ordering them by the lowest mean rank. Ties between genes with the same average adjusted *P*-value rank were broken by ranking the genes based on mean logFC rank. The following genes were culled from the list due to their inability to be tested via available RNAi lines; mitochondrial genes, pseudogenes designated by CR#, asRNA, hpRNA, snRNA, snRNP, snmRNA, snoRNA, lncRNA, sisRNA, and pre-rRNA. Ribosomal structural genes were also removed due to their ubiquitous expression and function. The top 500 ranked germline genes were then taken as candidate genes.

Gene Ontology over-representation analysis was run on the candidate genes against a background list of all expressed genes using clusterProfiler (v4.6.2; R 4.2.3) to find enriched Biological Process terms (Ashburner *et al*. 2000; Gene Ontology Consortium *et al*. 2023).

### Drosophila stocks and genetics

All Drosophila RNAi stocks utilized were created by the Harvard Transgenic RNAi project (TRiP collection) (Zirin *et al*. 2020) and ordered from the Bloomington Drosophila Stock Center (BDSC) as of November 2022 (Supplementary Table 3). Germline TRiP lines (VALIUM20, VALIUM21, and VALIUM22 vectors) targeting genes ranked in the top 500 of the scRNA-seq list were screened. All lines were screened when a gene had multiple germline TRiP lines available. There were 331 genes with at least one germline TRiP line, 140 of which had multiple lines available, for a total of 526 lines screened (Table 1 and Supplementary Table 3). Full information for these lines can be found on the TRiP website (https://fgr.hms.harvard.edu/trip-in-vivo-fly-rnai).

**Table 1.** Summary of the top 500 list.

	Number
Lines	
Lines with underdeveloped ovaries	74
Lines with phenotypes in the germaria*^a^*	84
Lines with wild-type germaria	368
Lines tested	526
Genes	
Genes with a line causing underdeveloped ovaries	51
Genes with a line causing phenotypes in the germaria*^a,b^*	61
Genes with all lines displaying wild-type germaria	222
Genes tested	331
Genes with no germline TRiP	169
*CG* designated genes*^c^*	
*CG* designated genes*^d^*	154
*CG* designated genes with no germline TRiP	93
*CG* designated genes with underdeveloped ovaries	1
*CG* designated genes causing a phenotype in the germaria*^b^*	5

^*a*^Phenotypes affecting development, SC, or DSBs in the germarium.

^*b*^Two genes had at least one line causing underdeveloped ovaries and at least one line causing a phenotype in germaria.

^*c*^ *CG* genes are included in the gene categories.

^*d*^Lines with only *CG* designations in FlyBase as of November 2022 (Gramates *et al*. 2022).

Gene names provided in Supplementary Tables 3 and 4 were those listed in FlyBase (https://flybase.org) as of November 2022 (Gramates *et al*. 2022). Short descriptions of the genes provided in Supplementary Table 3 were derived from FlyBase and may not cover all functions for genes involved in multiple biological processes (Gramates *et al*. 2022). Bloomington lines 36303 (*y v; attP2, y+*) and 36304 (*y v; attP40, y+*) were used as controls. The germline-specific *nanos-GAL4* driver (*y w; nanos-GAL4::VP16; spa^pol^*) was used to express UAS-RNAi lines (Rørth 1998). *UAS-RNAi* males from each TRiP line were crossed to *nanos-GAL4* virgin females. One-to-three-day-old adult female progeny heterozygous for both *nanos-GAL4* and the *UAS-RNAi* was transferred to a new vial and held overnight with males and wet yeast paste before being dissected for cytological preparations.

### Immunostaining of whole-mount adult ovaries

Germarium preparations for whole-mount immunofluorescence were performed as described by Lake *et al*. (2015), with the following exceptions. Dissections were performed in PBS with 0.1% Tween 20 (PBST). For some preparations, ovaries were blocked overnight at 4 °C while nutating in PBST with 1% bovine serum albumin (BSA) (EMD Chemicals, San Diego, CA).

### Larval ovary dissection and immunostaining

*w^1118^hyb5b* flies were raised on standard food at 25 °C. *w^1118^hyb5b* is a partially isogenized *w^1118^* stock. Wandering third instar larvae were collected, dissected, and immunostained as in Maimon and Gilboa (2011), with the following modifications. After removing the head, the larvae were held at the posterior end by a pair of forceps while a second pair of forceps held laterally was used to gently squeeze the larval tissues through the apical opening. Distal fat bodies containing the larval ovaries were separated from the other tissues and placed in an ice-cold Ringer's medium in a cell strainer using a large bore pipette tip. Samples were mounted in 28 μL of Prolong Glass.

### Primary and secondary antibodies

Primary antibodies used for adult ovary preparations were mouse anti-C(3)G 1A8-IG2 [1:500] (Page and Hawley 2001), rabbit anti-histone γH2AVD pS137 [1:5000] (Rockland Inc., Lots 41635 and 46042), and mouse anti-Orb 6HA [1:40] (Developmental Studies Hybridoma Bank, Iowa) (Lantz *et al*. 1994). For larval ovaries, mouse anti-C(3)G 1A8-IG2 [1:500] (Page and Hawley 2001), rabbit anti-Corolla [1:2000] (Collins *et al*. 2014), and rat anti-Vasa [1:25] (Hay *et al*. 1990) were used. We used the following goat Alexa Fluor secondary antibodies at [1:500]: anti-mouse 488 or 555, anti-mouse IgG1 488 or 555, anti-mouse IgG2A 555 or 647, anti-rat 647, and anti-rabbit 488 or 647 (ThermoFisher).

### Imaging and image analysis

Images of adult ovaries were acquired on an inverted DeltaVision microscopy system (GE Healthcare) with an Olympus 100× Objective (UPlanSApo 100× NA 1.40) and a high-resolution CCD camera. Images were deconvolved, full or partially projected from larger z-stacks, and cropped using SoftWoRx v. 7.1 software (Applied Precision/GE Healthcare). For improved publication, contrast was uniformly enhanced for some channels.

Images of larval ovaries were acquired with an Orca Flash 4.0 sCMOS 100fps at full resolution on a Nikon Eclipse Ti2 microscope equipped with a Yokagawa CSU W1 10,000 rpm Spinning Disk Confocal with 50 μm pinholes. Samples were illuminated with 405 nm (3.9 mw), 488 nm (8.5 mw), 561 nm (6.1 mw), and 640 nm (46 mw) lasers (LUNV 6-line Laser Launch) with nominal power measures at the objective focal plane. This spinning disk confocal is equipped with a quad filter for excitation with 405/488/561/640. Emission filters used to acquire this image were DAPI: 430–480 nm, GFP: 507–543 nm, red: 579–631 nm, and far-red: 669–741 nm. A Nikon Plan Apochromat Lambda 100× oil objective lens, N.A. 1.49, 0.065 μm/px was used to acquire the images with varying exposure times. Image analysis and adjustments to brightness and contrast were done in Fiji.

### Initial scoring of phenotypes

Genes with germline-optimized TRiP lines were initially screened for defects in SC assembly, SC maintenance, DSB initiation, and DSB repair using antibodies recognizing C(3)G (SC) and γH2AV (DSB). Lines were identified for retest if (1) DSBs were absent in region 2A, (2) DSBs persisted in region 3, (3) full-length SC was not present in region 2A or too few nuclei with full-length SC were present, or (4) SC began to fragment or was absent by region 3.

### Secondary scoring of lines with potential meiotic phenotypes

Lines identified with potentially interesting SC or DSB defects during initial screening were rescreened by immunostaining ovaries using anti-C(3)G (SC), anti-γH2AV (DSB), and anti-Orb (oocyte/cyst development) antibodies. At least 10 germaria were scored by eye for proper SC assembly/maintenance, DSB initiation/repair, and Orb localization. Images were acquired and scored for lines that required additional analysis. Lines were considered defective if at least 50% of the scored ovaries showed a defect. For genes in the piRNA pathway or with well-studied developmental defects with multiple lines available, not all lines identified with defects in initial scoring underwent secondary screening with Orb antibody.

### Phenotype classification

**Fig. 1.**
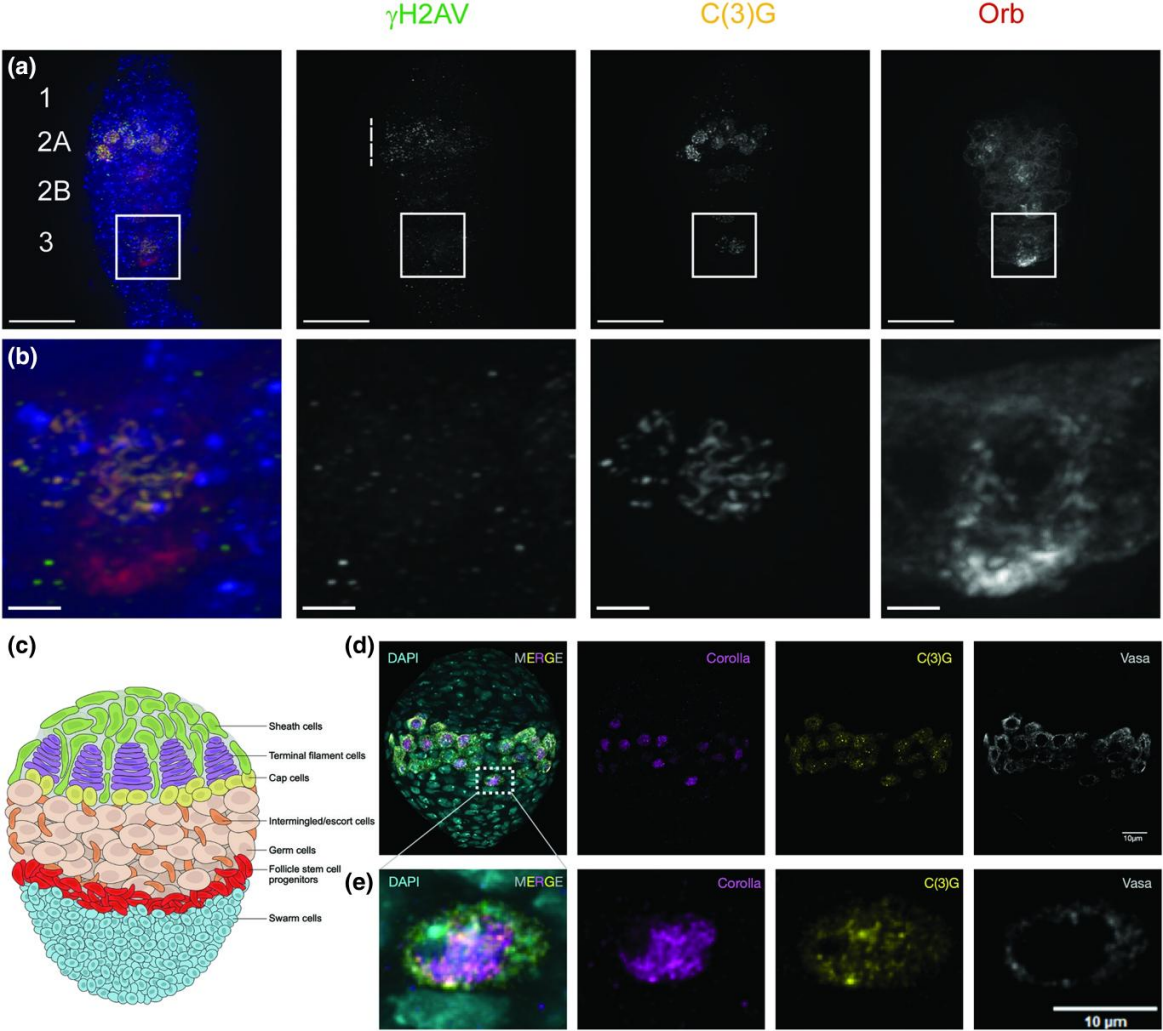
Markers of meiosis and development in the *Drosophila melanogaster* germarium. a) *attp 40* (BL36304)*/+*; *nanosGAL4/+* control germarium stained for antibodies against γH2AV to mark DSBs (green), C(3)G to visualize the SC (yellow), Orb to examine the 16-cell cysts and the nucleus specified to become the oocyte (dark red), and DAPI to visualize DNA (blue). Boxed region shown in (a) is enlarged in (b). During Drosophila ovarian development after the stem cell divides, the resulting daughter cell undergoes 4 additional rounds of mitosis with incomplete cytokinesis to form a 16-cell interconnected cyst [reviewed in Spradling (1993); McLaughlin and Bratu (2015)]. The cyst then enters meiosis visualized with the expression of diffuse Orb protein in the cyst. As the cyst progresses through the germarium, oocyte specification occurs visualized as an accumulation of Orb protein around the selected oocyte nucleus, and the 15 remaining cells become nurse cells that support the oocyte (Spradling 1993; Barr *et al*. 2019). Foci of SC proteins are first loaded near the centromeres during the mitotic divisions to form the 16-cell cyst in region 1 followed by additional SC initiation events at zygotene (Christophorou *et al*. 2013). Full-length SC is built between homologous chromosomes in multiple cells of the 16-cell cyst in region 2A (early pachytene). As the cyst progresses through the germarium, all but the specified oocyte nucleus disassembles its SC until only the Orb specified nucleus retains full-length SC at region 3 (boxed region). Double-stranded breaks (DSBs) first appear during early pachytene as visualized by γH2AV foci (dashed line) [reviewed in Hughes *et al*. (2018)]. γH2AV foci decrease in number as cyst development progresses signifying that DSB repair has been initiated with γH2AV foci mostly/entirely absent in the specified nucleus in region 3. Image is a projection from z-stack. Scale bars = 15 µm (a) and 2 µm (b). SC proteins are expressed in germ cells of the larval ovary. c) Schematic of a late-stage *Drosophila* larval ovary. d) Immunofluorescence images showing expression of vasa defining the central band of germ cells, broad Corolla expression in the germ cell nuclei, and C(3)G expression in both cytoplasm and nuclei with bright puncta restricted to germ cell nuclei. e) Magnified images of a single nucleus from panel (b). DAPI (cyan), Corolla (magenta), C(3)G (yellow), and vasa (gray). Scale bars: = 10 µm (d and e).

Those lines labeled as having developmental defects showed a complete lack of Orb staining or had one of the 4 phenotypes: (1) weak Orb staining in region 2A; (2) too few cysts in the germaria based on Orb staining; (3) failure of Orb to concentrate around a single pro-oocyte in region 2B/3; or (4) abnormal size and shape of cysts based on Orb staining (see Fig. 1, a and b for wild-type Orb localization) (Lantz *et al*. 1994). If the developmental defects were strong, such as Orb was not expressed or cyst arrangement was abnormal, the ovaries were not further analyzed in detail for SC and DSB phenotypes due to an inability to reliably identify stages of oocyte development.

Lines were classified as having SC assembly defects if no full-length SC was present in 2A or if only a few nuclei built full-length SC by mid-region 2A. Lines were classified as having an SC maintenance defect if full-length SC was absent in the posterior region 2B cyst and/or region 3 oocyte as marked by Orb (see Fig. 1, a and b for wild-type phenotypes). Lines that made SC aggregates in at least 50% of the images scored were classified as having polycomplexes. DSB initiation defects were assigned if no DSBs as marked by the phosphorylation of histone H2AV (γH2AV) were present in region 2A. A DSB repair defect was assigned if 2 or more γH2AV foci were present in the specified oocyte as marked by Orb in region 3.

## Results and discussion

### Identifying genes expressed in early meiotic/pachytene cells

To enrich for candidate genes required for oocyte development, SC formation, SC maintenance, and meiotic recombination in *Drosophila melanogaster* females, we took advantage of the availability of 2 recently published single-cell transcriptomic analyses of adult ovaries (Rust-adult, Slaidina-adult) as well as a third analysis of developing larval ovaries (Slaidina-larval) (Rust *et al*. 2020; Slaidina *et al*. 2020, 2021). We show in Fig. 1, c–e that the C(3)G and Corolla proteins are expressed in larval ovaries; therefore, the inclusion of this dataset could potentially enrich for transcripts specifically needed in early meiotic cells. We analyzed these datasets to try to identify genes expressed in cells undergoing early stages of meiosis (meiotic entry through pachytene).

**Fig. 2.**
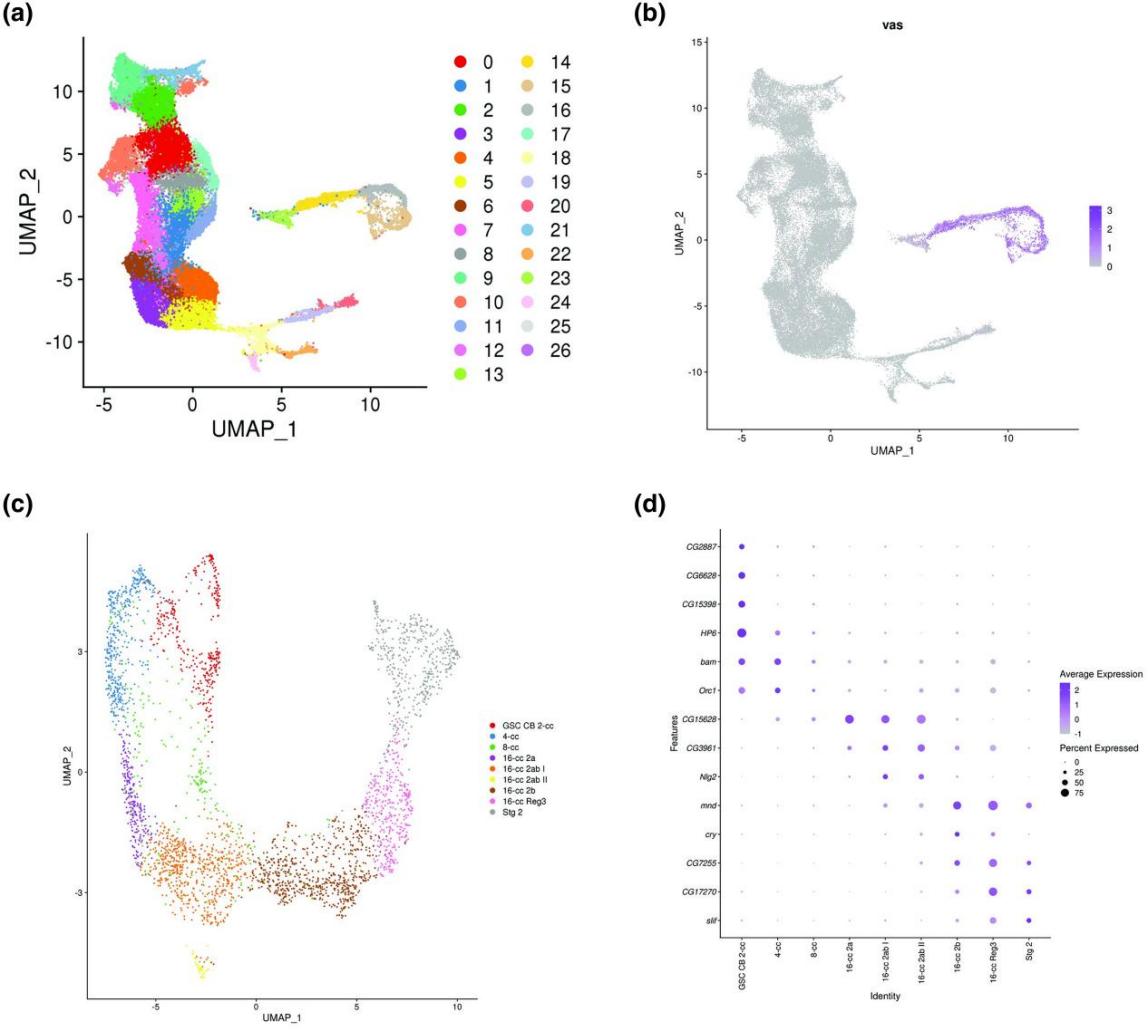
Identification of germline subclusters representing 9 cell types. a) UMAP from the 9 replicates integrated together. Somatic cell clusters (0–12 and 17–26) and germline cell clusters (13–16) are readily distinguished. b) Feature plot of the germline marker *vasa* showing specificity of the germline cluster. c) UMAP showing germ cell subclustering into 9 cell types found in the adult germarium and stage 2 oocytes. d) Dot plot showing expression of 14 marker genes [identified in Slaidina *et al*. (2021)] in our dataset. Dot color represents average normalized expression level and diameter represents fraction of cells expressing the gene in each cluster.

To find genes expressed in meiotic cells, the 3 single-cell datasets were reanalyzed both individually and integrated together to identify genes that co-expressed with 4 marker genes. Three of the marker genes were the SC central region genes, *c(3)G*, *corolla*, and *cona,* which should be expressed in cells that have entered the meiotic program. Studies have shown that these SC proteins are expressed as early as the mitotic divisions in region 1 of the germarium to generate the 16-cell interconnected cysts (Fig. 1a) (Christophorou *et al*. 2013). The gene *vasa* was also included as a marker gene since it is required for germ cell specification and is highly expressed in germ cells (Hay *et al*. 1988, 1990). This analysis resulted in a list of 2,739 genes that were co-expressed with the 4 marker genes for a total of 2,743 genes (Supplementary Table 4). Figure 2a shows a UMAP from our analysis of the 9 integrated replicates showing clean separation of germ cell and somatic cell clusters. Figure 2b is a feature map of *vasa* showing its specific expression within the germ cell cluster. To better understand our germline cell population, the germline cells in the integrated analysis were subsetted into 9 clusters using Seurat. These clusters were then assigned annotations based on the expression of germarium cell type-specific marker genes found in Slaidina *et al*. (2021) (Fig. 2, c and d).

In each analysis, genes of interest were found by comparing the distribution of gene expression levels within our germline cluster to the rest of the population. Only genes with increased levels of expression in the germline cells and an adjusted *P*-value of <0.05 (according to the Wilcoxon Rank Sum test with Bonferroni Correction) were considered. These co-expressed genes were ordered based on their adjusted *P*-values. After culling gene classes not easily tested due to a lack of available RNAi lines and ubiquitously expressed ribosomal RNA genes (see Materials and Methods), a final ranking was then applied by calculating the average rank of each gene across the multiple analyses. Genes not found as co-expressed genes in all 4 analyses were not included, giving us a final list of 2,743 genes (Supplementary Table 4). In the final ranking list, the 4 marker genes scored high with *cona* ranked 3rd, *vasa* 7th, *c(3)G* 10th, and *corolla* 20th.

**Fig. 3.**
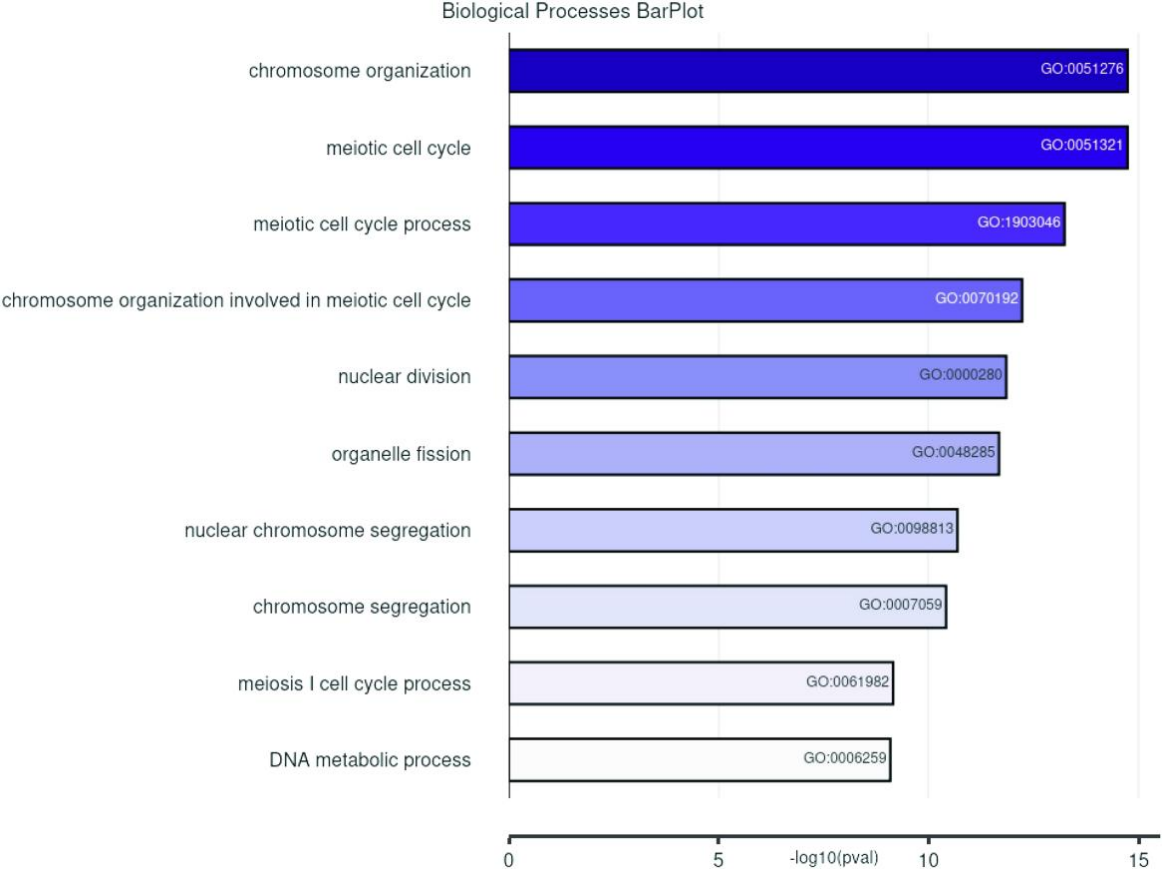
GO term analysis of top 500 genes. Barplots showing the −log10(pval) for the top 10 significant Biological Process Gene Ontology terms found using clusterProfiler on the list of top 500 ranked genes (Ashburner *et al*. 2000; Gene Ontology Consortium *et al*. 2023), https://bioconductor.org/packages/release/bioc/html/clusterProfiler.html.

With the overall high ranking of the marker genes, we chose to more closely examine the gene ontology of the top 500 ranking genes (Ashburner *et al*. 2000; Gene Ontology Consortium *et al*. 2023). This shortened list was enriched for genes involved in biological processes expected for cells in meiosis and ovarian development such as the meiotic cell cycle, chromosome organization, and chromosome segregation (Fig. 3). Indeed, the top 500 list contained genes for proteins of meiotic cohesin complexes and the lateral element [*ord*, *solo*, *sunn*, *c*(2)*M*, *SMC1*, *SMC3*]; multiple known regulators of oocyte development, including *ovo*, *orb*, *bam*, *mei-P26* and *otu*; as well as 5 recombination proteins (*vilya*, *narya*, *nenya, Mcm5,* and *hdm)* (Supplementary Tables 3 and 4). The high number of known meiosis and development genes present on the list indicated that examining the genes within the top 500 list would likely yield additional genes important for early oocyte development and meiosis.

### Functional tests of the highly ranked genes

To validate the list of genes from single-cell transcriptome data and to identify potentially new genes with roles in early meiotic cells, we examined how decreasing the transcript levels of the top 500 genes affected SC regulation, recombination, and oocyte development. Germline expressed RNAi hairpins with GAL4-responsive upstream activating sequences were driven by the *PnanosGAL4::VP16* driver (referred to as *nanosGAL4*). The *nanosGAL4* driver has GAL4 under the control of *nanos* regulatory elements, which robustly expresses transcripts with UAS sequences in the stem cells and meiotic cells of the early ovary (Rørth 1998). This limits the knockdown of gene expression, specifically within the germline but not the somatic support cells of the ovary. Hairpin lines tested were those available at the Bloomington Stock Center from the Harvard Transgenic RNAi Project (TRiP) as of November 2021.

Two of the advantages of this strategy were that at least one germline expressed RNAi hairpin was available for 331 of the top 500 genes and that knockdown could be assessed in a single generation (Table 1, Supplementary Table 3). Additionally, this approach circumvented the issue of genetic mutations potentially causing lethality during the development of the organism. Lines available for genes with known roles in meiosis (including the marker genes) and oocyte development were still included in the analysis to assess the level of efficacy of available TRiP RNAi lines. As RNAi can lead to only a partial knockdown of expression, examination of known meiotic and development genes also had the potential to reveal new phenotypes that may have not been observed due to lethality or earlier developmental phenotypes of strong loss-of-function mutations. We tested 526 RNAi lines that covered a total of 331 of the top 500 genes (Table 1, Supplementary Table 3).

Ovaries from flies expressing RNAi lines driven by *nanosGAL4* were first examined by immunofluorescence for SC formation and maintenance using antibodies recognizing C(3)G, the transverse filament protein of the SC, and γH2AV, the phosphorylation mark present on H2AV at DSB sites (Fig. 1a) (Page and Hawley 2001; Mehrotra and McKim 2006; Lake *et al*. 2013). Foci of the C(3)G form near the centromeres in multiple nuclei during the mitotic divisions to make the 16-cell cyst in region 1 followed by initiation of additional patches of SC formation at zygotene (Fig. 1a) (Christophorou *et al*. 2013). In region 2A (early pachytene), up to 4 cells of the cyst will undergo SC elongation to form full-length tracks of SC between the homologs. DSBs form soon after full-length SC formation, visualized as foci of γH2AV. As the cyst progresses to regions 2B (early-mid-pachytene) and 3 (mid-pachytene) of the germarium, the full-length SC is disassembled from all but one nucleus within the cyst, which will become the oocyte. Repair of the DSBs is also initiated, which can be observed as a decrease in the number of γH2AV foci in region 2B and few, if any, γH2AV foci in region 3 (Fig. 1, a and b).

After the initial characterization, lines were kept for further analysis if full-length tract-like SC was not observed in region 2A or if too few cells initiated full-length SC formation (see Materials and Methods for full details for scoring). Lines were scored positive for a defect in SC maintenance if the SC in the single oocyte nucleus in region 3 had begun to fragment or was absent. Defects in DSB dynamics would be noted if γH2AV foci were absent in regions 2A and 2B (an indication of a failure in meiotic DSB formation) or if numerous γH2AV foci were observed in region 3 (an indication of a possible failure or delay in meiotic DSB repair). RNAi lines that were identified as possibly having defects underwent a secondary screening by staining with an antibody recognizing Orb to test for defects in development that may be the underlying cause of SC and DSB phenotypes. Orb is required to specify oocyte fate and the localization pattern can provide indications of defects in cyst development and oocyte specification (Barr *et al*. 2019). Orb localization diffuses within the cytoplasm of the entire cyst just after meiotic entry when full-length SC is present (Lantz *et al*. 1992, 1994). As the cyst progresses through the germarium, Orb protein becomes increasingly concentrated around the nucleus that will be specified as the oocyte nucleus but remains in a weak haze around the nurse cell nuclei in the cyst (Fig. 1, a and b). The absence of or mislocalized Orb protein indicates that the targeted gene has a role in oocyte development and that observed defects in SC and DSB progression are likely indirectly caused by failures to properly form the cysts and specify the future oocyte.

### RNAi knockdown of gene transcripts in the top 500 list caused SC, DSB, and developmental phenotypes

**Fig. 4.**
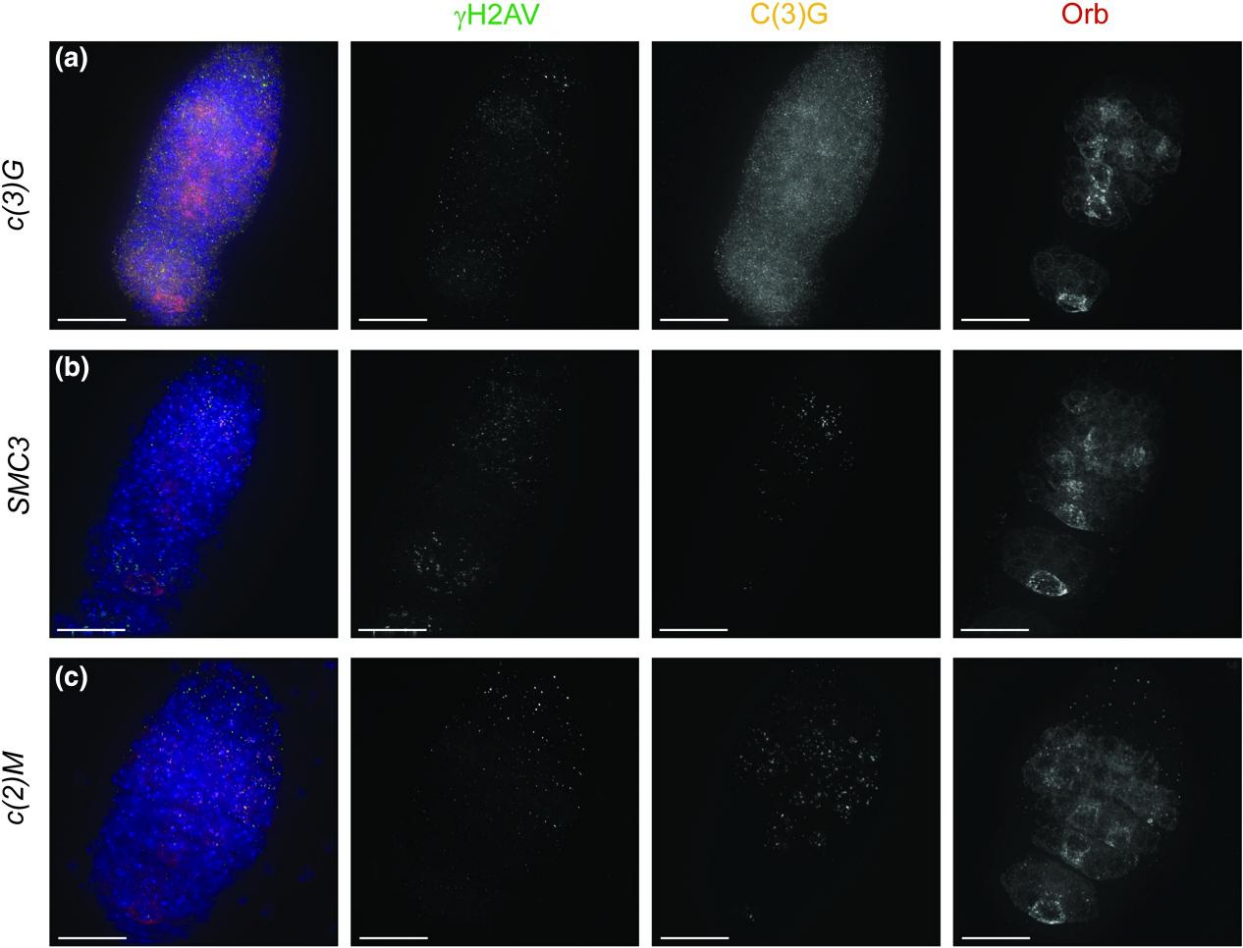
SC phenotypes with wild-type Orb localization can be observed in RNAi lines targeting known components of the SC central region, lateral element, or cohesin complexes. Shown are antibodies recognizing γH2AV (DSBs in green), C(3)G (SC in orange), and Orb (cyst development and oocyte specification in dark red). DAPI (DNA) is in blue. All lines are driven with *nanosGAL4* a) RNAI knockdown of the gene encoding the transverse filament protein of the SC *c(3)G* (BL62969) results in the absence of any SC formation but Orb and γH2AV show wild-type dynamics. b) RNAi knockdown of *SMC3* (BL50899), a core cohesin component, leads to only puncta of C(3)G but Orb and γH2AV show wild-type dynamics. c) Knockdown of the lateral element protein *c*(2)*M* (BL43977) results in puncta of C(3)G, but Orb and γH2AV show wild-type dynamics. Images are projections from z-stacks. Scale bars = 15 µm.

Since the level of knockdown of mRNA transcripts varies between RNAi lines, we examined the phenotypes caused by all available TRiP lines for known meiotic genes in the top 500 to assess the effectiveness of this strategy to identify genes with roles in the early ovary. Besides the central region components of the SC [*cona*, *corolla*, and *c(3)G*] that were used as our markers for early meiotic cells, the following known SC-related genes of the lateral element and cohesin complexes were also present in our top 500: *c*(2)*M, SMC1, SMC3, sunn, solo*, *ord*, and *Nipped-B.* RNAi lines targeting *c(3)G*, *corolla*, *c*(2)*M*, *Nipped-B*, and 3 of the lines targeting *SMC3* caused strong defects in full-length SC assembly consistent with prior studies of genomic alleles and RNAi lines, illustrating RNAi lines can be used to assess SC formation defects (Supplementary Table 3, Fig. 4) (Page and Hawley 2001; Manheim and McKim 2003; Collins *et al*. 2014; Gyuricza *et al*. 2016). However, RNAi lines targeting *SMC1*, *sunn*, *ord*, and an additional *SMC3* line failed to produce an SC phenotype in the germarium in our assay, which is approximately one-third of the assayed lines targeting the known SC-related genes.

Restricting our analysis to the top 500 genes identified only 5 genes (*vilya*, *narya*, *nenya*, *Mcm5,* and *hdm*) encoding proteins with known functions in recombination (see below). Of the 3 genes with available lines (*Mcm5*, *hdm*, and *narya*), only the *Mcm5* lines had a phenotype in our assays (Supplementary Table 3). These results demonstrate that although this is a useful screening method, using the RNAi screening strategy would likely miss some proteins with roles during early meiosis. Additionally, some of the known meiotic genes (for example, *cona* and *solo*) had no lines available to test at the Bloomington Stock Center, which would represent another class of potentially missed genes.

**Fig. 5.**
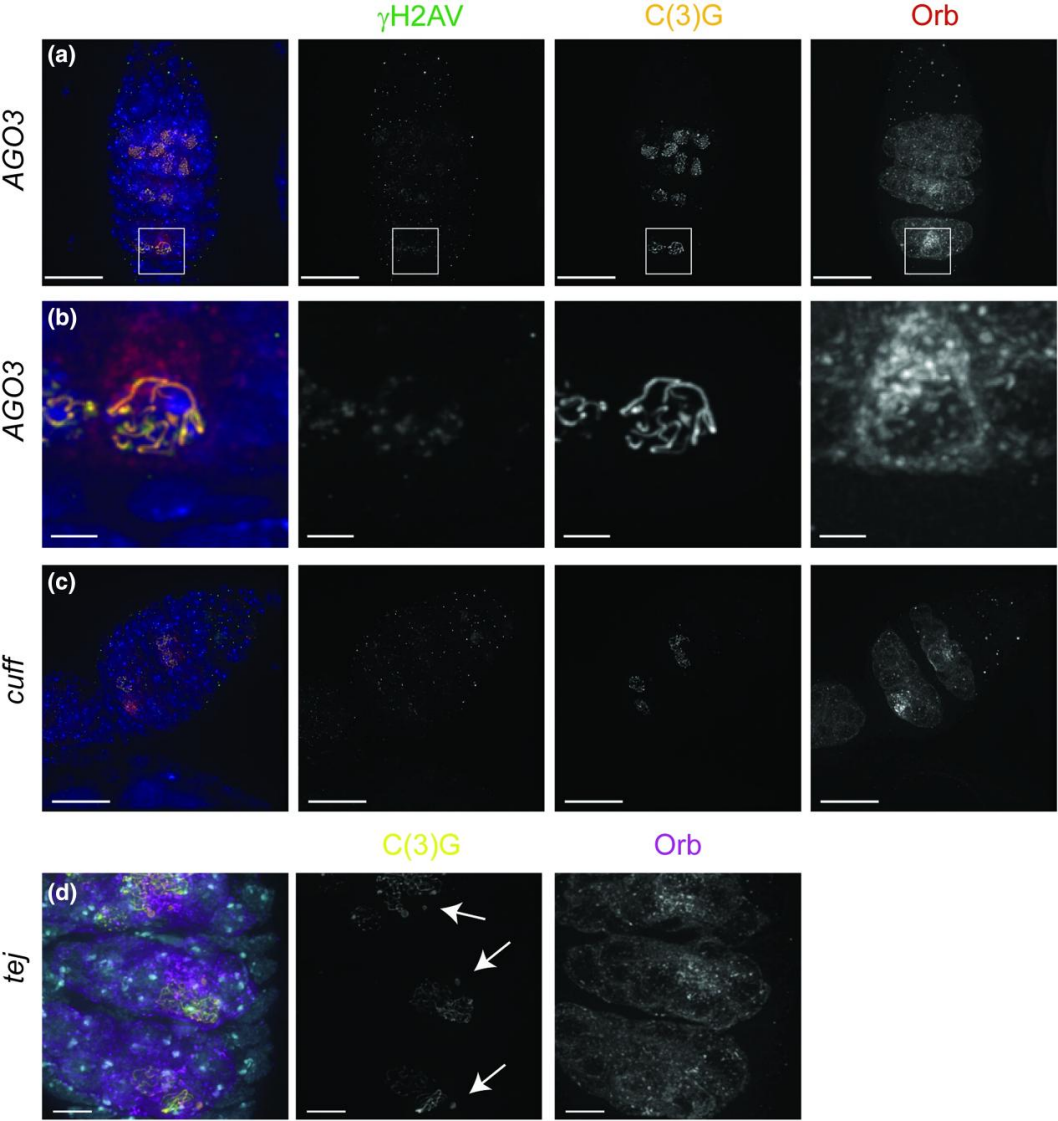
RNAi knockdown of genes in the piRNA pathway cause differing phenotypes. a) and b) γH2AV foci are present in the region 3 oocyte nucleus in a germarium expressing AGO3 RNAi (BL44543). Boxed region shown in (a) is enlarged in (b). c) Germarium expressing cuff RNAi (BL35318) displaying a delay in cyst development. a–c) Germarium were stained for antibodies against γH2AV to mark DSBs (green), C(3)G to visualize the SC (yellow), Orb to label cysts (dark red), and DAPI to visualize DNA (blue). d) Knockdown of the piRNA *tej* causes the formation of small polycomplexes. In *tej* RNAi (BL36875) full-length SC forms but additional small polycomplexes can be observed (arrows). SC is labeled with C(3)G (yellow), cysts are labeled with Orb (magenta), and DNA is labeled with DAPI (cyan). RNAi lines were driven with *nanosGAL4*. Images are projection from z-stacks. Scale bar = 15 µm in (a) and (c), 2 µm in insets of region 3 (b), and 5 µm in (d).

The top 500 genes included many of the genes of the piRNA pathway, which allowed us to examine the effectiveness of using γH2AV as a scoring marker. One role of the piRNA pathway is to repress the mobilization of transposable elements in the germline (Huang *et al*. 2017; Czech *et al*. 2018; Sato and Siomi 2020). Loss of piRNA genes results in transposon mobilization events that can be visualized as γH2AV-marked DSBs in the selected region 3 oocyte nucleus (Fig. 5, a and b). This phenotype would look similar to mutants with failed or delayed repair of preprogrammed meiotic DSBs (Mehrotra and McKim 2006). Multiple lines targeting piRNA genes tested positive for the scoring criteria of multiple γH2AV foci present in the region 3 oocyte nuclei (Supplementary Table 3). Figure 5, a and b shows an example of γH2AV foci in a region 3 oocyte nucleus observed in an ovary expressing an RNAi construct targeting *AGO3*. Several piRNA lines also showed developmental defects. Underdeveloped ovaries were observed for 2 lines targeting *piwi* (Supplementary Table 3), which is consistent with genetic mutations (Lin and Spradling 1997; Cox *et al*. 1998), and fewer nuclei with SC due to delays in Orb localization to cysts occurred when *cuff* was targeted (Fig. 5c). Germline knockdown of *tej* resulted in the formation of small SC polycomplexes similar to those reported for *aub* mutants (Blumenstiel *et al*. 2008) (Fig. 5d). The range of germline phenotypes observed for lines targeting piRNA proteins illustrates their many roles in the early Drosophila germline and demonstrates that screening RNAi lines cytologically can reveal a range of phenotypes.

**Fig. 6.**
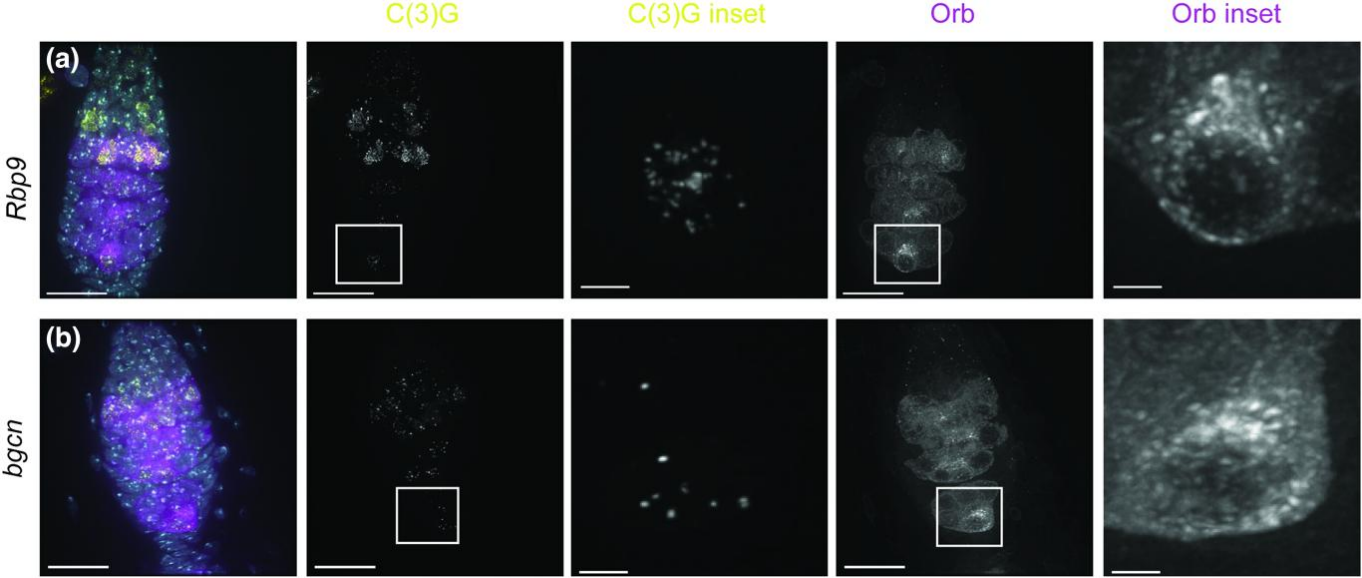
RNAi knockdown causes hypomorphic phenotypes for genes required for oocyte development. RNAi knockdown of genes required for oocyte development cause SC phenotypes despite the production of cysts with Orb localization and Orb specifying a nucleus in region 3. Genes targeted are a) *Rbp9* RNAi (BL42796) and b) *bgcn* (BL36636). SC is labeled with C(3)G (yellow), cysts are labeled with Orb (magenta), and DNA is labeled with DAPI (cyan). Insets show magnification of the boxed region. Scale bar = 15 µm in germaria. Scale bar = 2 µm (insets). RNAi lines were driven with the *nanosGAL4* driver. Images are full (germaria) or partial (inset) projections of z-stacks.

The list of the top 500 genes also included many genes with essential roles in early oocyte development, including *orb*, *otu*, and *bam,* which all had RNAi lines that caused underdeveloped ovaries when expressed with *nanosGAL4* consistent with phenotypes of strong loss-of-function mutants (McKearin and Spradling 1990; Geyer *et al*. 1993; Lantz *et al*. 1994). Hypomorphic phenotypes could also be observed. While null mutations of *Rbp9*, which regulates *bam* expression, have been reported to cause severe ovarian developmental defects (Kim-Ha *et al*. 1999), germline knockdown of *Rbp9* by RNAi resulted in germaria displaying only a mild delay in the appearance of Orb protein and Orb still accumulated around a single nucleus in region 3, indicating some aspects of oocyte specification was initiated (Fig. 6a). In 12/15 germaria, at least 2 nuclei with full-length SC could be observed in region 2A, but then the SC quickly fragmented by region 2B (Fig. 6a). An interesting hypomorphic phenotype was also observed for the line targeting *bgcn*. Null mutations of *bgcn* are reported to cause a tumorous germline due to a failure in proper differentiation of the germline stem cell (Geyer *et al*. 1993). In germaria with *bgcn* knockdown, early cyst development appears to occur normally based on Orb localization and DNA morphology (Fig. 6b). Despite the formation of cysts with Orb accumulation, full-length SC fails to form with only puncta of SC proteins in all cysts (Fig. 6b). These likely hypomorphic conditions could prove useful for investigating the interaction between oocyte specification and SC regulation. Additionally, these results indicate that proteins like Rbp9 play indirect roles in maintaining SC, which would have been missed examining flies with null mutations.

### Many genes expressed during early meiosis regulate early oocyte development

Of the 331 genes tested in the RNAi screen, 51 had at least one line that caused the formation of ovaries too underdeveloped to analyze cytologically using our methods. Therefore, approximately 15% of the tested genes resulted in strong developmental defects, indicating the top 500 list was enriched for genes required for critical processes early in ovarian development (Table 1, Supplementary Table 3). This included one uncharacterized gene, *CG8142*, which is predicted to be part of Elg1 RFC-like complex by FlyBase (Gramates *et al*. 2022). The observation that knockdown of the unknown gene *CG8142* causes defects in the earliest steps of ovarian development indicates that there are still unidentified players even in the early steps of cyst formation.

**Fig. 7.**
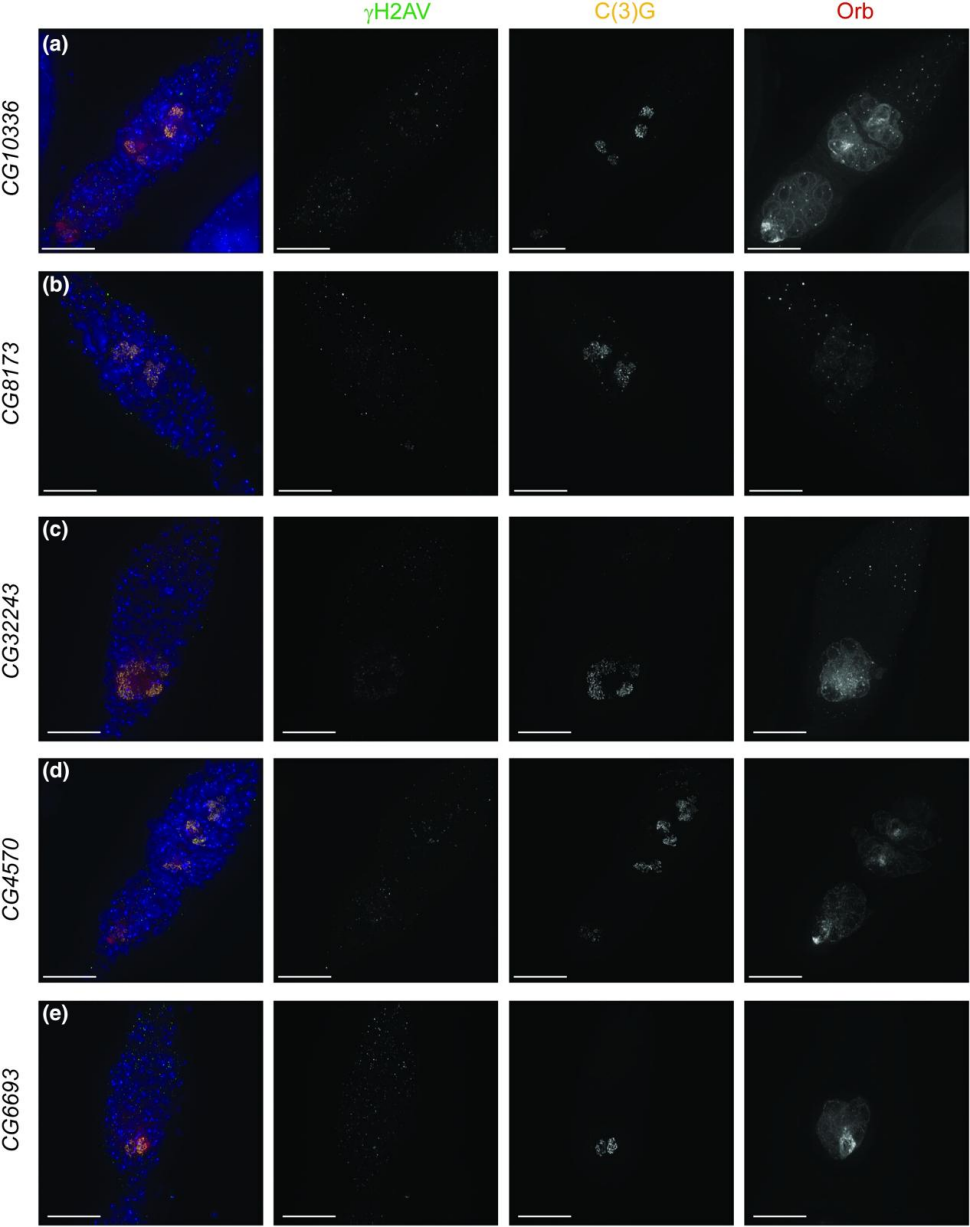
RNAi knockdown of uncharacterized genes causes delays or reductions in Orb positive cysts as well as delays in meiotic events. Shown are antibodies recognizing γH2AV (DSBs in green), C(3)G (SC in yellow), and Orb (cyst development and oocyte specification in dark red). DAPI (DNA) is in blue. All lines are driven with *nanosGAL4*. a) CG10336 (BL44459), b) CG8173 (BL51738), c) CG32243 (BL36082), d) CG4570 (BL57378), and e) CG6693 (BL60420). Images are projections from z-stacks. Scale bars = 15 µm.

The initial screen yielded numerous genes that when targeted by RNAi caused a strong reduction in the number of nuclei with SC or a delay in full-length SC formation until region 2B or 3 (Fig. 7, Supplementary Table 3). Our secondary screening looking at Orb localization revealed developmental defects for many of the genes. Five uncharacterized genes were found to have such developmental issues, *CG10336* (predicted to be important for cell survival after DNA damage or replication stress), *CG8173* (orthologous to human *PDZ binding kinase*), *CG32243* (no predicted function), *CG4570* (predicted to enable nucleic acid binding activity), and *CG6693* (orthologous to human *DnaJ heat shock protein family Hsp40 member C9*) (Fig. 7). All displayed a decrease in the number of cysts with Orb localization and/or delays in the accumulation of Orb protein in the 16-cell cysts in multiple germaria, sometimes as late as region 3. Full-length SC formation was delayed until Orb protein began to accumulate in the cysts. A failure of Orb to sufficiently accumulate in the cysts and nuclei displaying only punctate/highly fragmented SC rather than full-length SC was observed in 4/10 *CG32243*, 3/13 *CG6693*, and 7/12 *CG8173* targeted germaria. Future studies will be required to determine how exactly these proteins function in regulating ovarian development.

### Lines exhibiting delayed SC assembly and DSB initiation

**Fig. 8.**
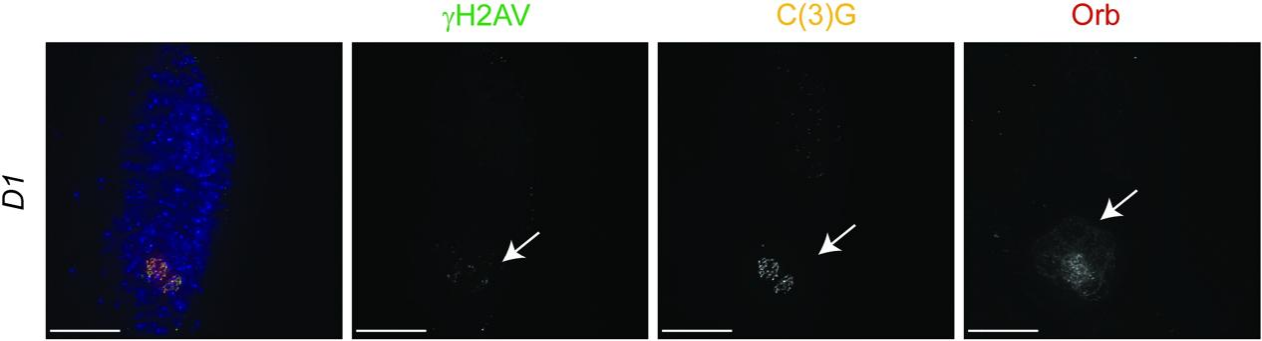
Germline knockdown of *D1* causes delays in development and meiosis. Shown are antibodies labeling γH2AV (DSBs in green), C(3)G (SC in yellow), and Orb (cyst development and oocyte specification in dark red). DAPI (DNA) is in blue. All lines are driven with *nanosGAL4*. *D1* RNAi (BL33655) causes delays in cyst development, SC formation, and DSB formation. Arrows point to the SC formation, DSBs, and Orb expression that initiates at the posterior of the germarium where the region 3 oocyte would normally be located. Images are projections from z-stacks. Scale bars = 15 µm.

Delayed SC assembly was observed for lines targeting genes that have been more extensively studied in somatic cells. The germaria were found to also have developmental defects, for example, the heterochromatin binding protein D1 (Fig. 8). Knockdown of *D1* caused delays in Orb expression. In 4/10 germaria, the Orb expression was delayed until the posterior of the germarium in a more region 3 shaped cyst. Even when robust Orb expression commenced at region 3, full-length SC formed in 3 of the germaria (with the fourth displaying fragmented SC). γH2AV foci were also delayed until full-length SC formation, indicating the timing of the recombination program can be shifted when there are developmental delays.

While region 2A is the normal commencement of meiotic entry with full-length SC formation and DSB induction, the lines delaying Orb localization indicates some flexibility in meiotic timing. Cysts still had the potential to initiate meiotic events even when the normal timing of meiotic entry was missed within the germarium. This shift in meiotic timing could provide insight into the exact mechanisms that signal meiotic entry and the minimal requirement needed to trigger full-length SC formation and DSB induction. It will also be of interest to examine how shifting the timing of meiotic events affects later events of meiotic progression and oocyte development. Even in those lines showing strong Orb delays, there is a possibility that these genes could play additional roles directly in SC and DSB regulation, but it would require different knockdown conditions to better examine these potential roles.

### Knockdown of *mnb* caused a dramatic fragmentation defect

**Fig. 9.**
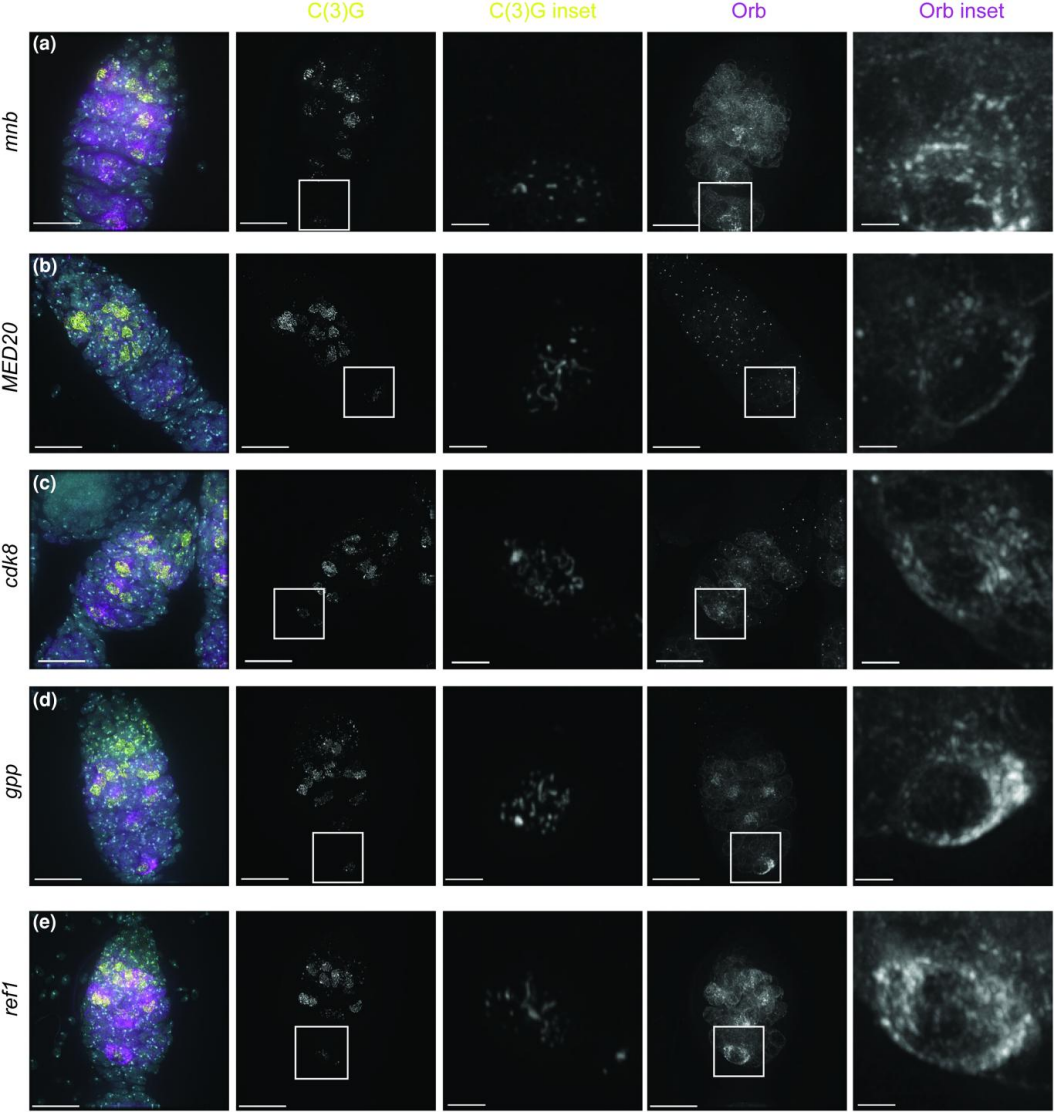
Germline knockdown of genes cause early disassembly of the SC. SC disassembly by region 3 is observed for genes that affect different processes. a) *mnb* RNAi (BL35222), b) *MED20* RNAi (BL34577), c) *cdk8* (BL35324), d) *gpp* RNAi (BL34842), and e) *ref1* RNAi (BL34626). SC is labeled with C(3)G yellow, Cysts are labeled with Orb (magenta), and DAPI labeled with DNA (cyan). Insets show magnification of the boxed region. Scale bar = 15 µm in germaria. Scale bar = 2 µm insets of region 3. All RNAi lines were driven with the *nanosGAL4* driver. Images are full (germaria) or partial (inset) projections of z-stacks.

We observed that knockdown of *mnb*, a Ser/Thr kinase with well-established roles in somatic brain and neuronal cell proliferation and differentiation, caused a meiotic phenotype (Tejedor *et al*. 1995; Shaikh *et al*. 2016). Germline knockdown of *mnb* displayed a strong early SC fragmentation phenotype even with accumulation of Orb protein in region 3 (Fig. 9a). It was previously reported that half of eggs from females expressing the *mnb* RNAi construct with the ovarian maternal triple driver failed to hatch, but the mechanistic cause of the decrease was not described (Sopko *et al*. 2014). Examination of an endogenously expressed GFP-tagged Mnb protein revealed that Mnb protein is primarily expressed in early region 2 of the germarium (Jevitt *et al*. 2020). Whether *mnb* acts in a similar mechanistic role in the ovary as it does in somatic tissues requires further investigation. The new ovarian phenotypes of genes better characterized in the soma indicate that there may be additional genes that play unknown roles in ovarian development and meiotic progression that may have been missed due to traditional mutants not surviving to adulthood.

### Genes that cause early disassembly of the SC

There were several lines with early SC disassembly in germaria that had SC formation and Orb expression in region 2A. Targeting *MED20*, a component of the Mediator complex, showed a strong early SC disassembly defect. Germaria were observed with full length SC in region 2A, but then the SC disassembled by region 3 and in some cases began to disassemble by region 2B (Fig. 9b). *MED20* was recently identified in another RNAi screen to have karysome defects in mid/late prophase oocytes (Nieken *et al*. 2023). It has been previously shown that mutations in chromatin modifiers that disrupt DNA structure also display SC defects (Zhaunova *et al*. 2016), suggesting that the early SC fragmentation caused by *MED20* knockdown may be due to defects in DNA structure (Fig. 9b). Knockdown of another component of the Mediator complex, *Cdk8*, caused a less severe early SC fragmentation phenotype with fragmentation occurring mostly in region 3 (Fig. 9c, Supplementary Table 3). *Cdk8* may also impact SC maintenance through regulating the DNA structure.

Early SC fragmentation was also observed for knockdown of several other lines, for example, those targeting *gpp* and *Ref1* (Fig. 9, d and e, Supplementary Table 3). A recent RNAi analysis of poorly characterized genes reported that RNAi knockdown of *Ref1* led to decreased female fertility which could at least be partially caused by the premature SC fragmentation phenotype we observed (Rocha *et al*. 2023). Further analysis will be needed to determine whether the targeted genes that cause SC maintenance defects regulate SC disassembly indirectly by regulating DNA structure in the oocyte or oocyte fate or whether these genes play more direct roles in maintaining full-length SC until mid-pachytene.

### Success and limitations of the screening strategy

The aim of the screen was to identify new genes that act in similar processes and in specific cell types focusing on genes highly co-expressed with our genes of interest. To discover new genes that function at the time of meiotic entry through early pachytene, we reanalyzed 3 published single cell datasets to create the list of the top 500 genes expressed with the germ cell marker *vasa* and 3 SC genes (Rust *et al*. 2020; Slaidina *et al*. 2020, 2021). We then assessed effects on meiosis by RNAi knockdown of genes with available germline expressing TRiP lines for the top 500 genes of the 2,743 identified. Our screen identified 6 uncharacterized genes with roles in early development, which indicates this approach can be used to find new genes with roles at a specific developmental time and cell type. Additionally, new phenotypes affecting ovarian development and early meiosis were described for genes with roles previously better characterized in mitotic cells.

With 3 SC genes used to generate the top 500 list, the hope was to identify not only development genes but also new genes with direct roles in SC formation and recombination. None of the tested RNAi lines targeting unknown genes caused phenotypes similar to known SC-related genes (see Fig. 4) or failed to initiate DSB formation when development appeared normal.

There were several limitations in our screen design that may have prevented us from identifying new genes directly regulating SC formation and meiotic recombination. First, of the top 500 genes, 169 did not have an available RNAi line, and of those, 93 were uncharacterized genes with only a *CG* designation at the time of screening. We found developmental phenotypes associated with 6 of the 61 screened *CG* designated genes, suggesting that using other strategies such as creating RNAi lines or CRISPR-induced mutations in these 93 genes might yield additional genes with roles in early development and meiosis.

Second, while the use of the already available germline expressed RNAi hairpins allows for rapid screening of candidate genes, our analysis of lines with known meiotic phenotypes indicated that roughly a third of RNAi lines fail to knockdown transcript levels to a degree sufficient to cause phenotypes in our assay. It is possible that some of the 55 uncharacterized genes in the top 500 with available lines that appeared wild-type in our assay may still play a role in early meiotic and developmental events if additional RNAi lines or CRISPR-induced mutations were tested.

Third, the *nanosGAL4* driver drives strong expression of constructs in the mitotic divisions of the developing ovarian cysts (Rørth 1998). Those genes with RNAi lines causing underdeveloped ovaries due to the knockdown of transcripts in the mitotic divisions may still play additional roles later in ovarian development or in meiosis. More specific drivers with expression limited to after the mitotic divisions would be required to allow for analysis of these lines specifically during the period of full-length SC formation.

Off-target effects of RNAi lines are always a possibility that must be considered. While the Transgenic RNAi Project was designed to try to avoid off-targets, there is still a potential for second-site targets, especially for genes that are duplicated or are part of gene families. Examination of those lines with previously unreported germline phenotypes using additional reagents will be needed for future studies.

It is possible that all the core components of the SC central region and lateral element have been identified or the remaining SC components to be identified have redundant functions. Redundancy was observed for the SC proteins SYP-5 and SYP-6 in *C. elegans* (Hurlock *et al*. 2020). Until the *Drosophila* SC can be reconstituted in vitro, it will be unclear whether we have identified all the proteins of the *Drosophila* SC.

**Fig. 10.**
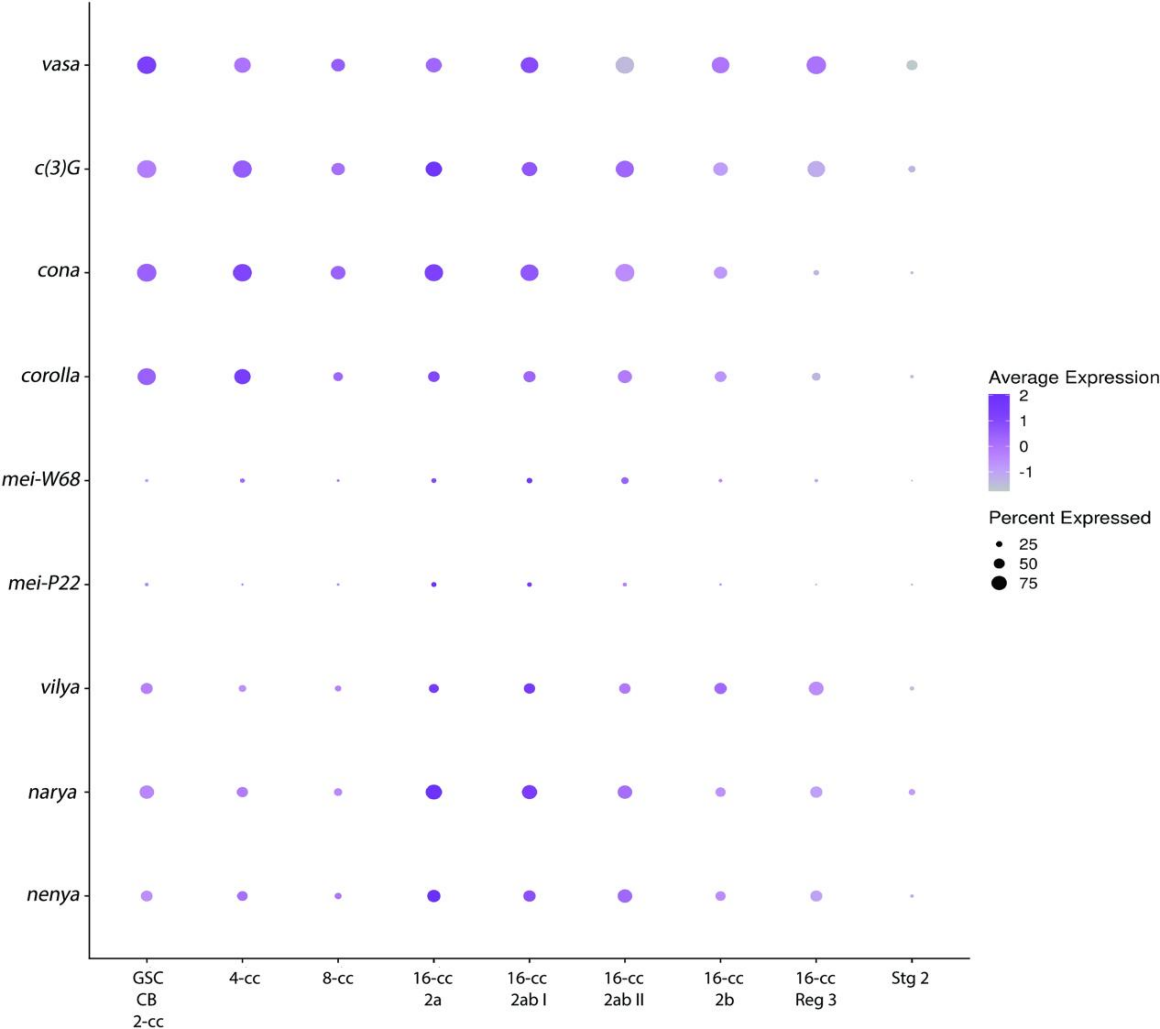
Expression dot plot comparing marker genes and recombination genes. Our selected marker genes have high expression in most cells across the germarium. Recombination genes identified in the screen, *vilya*, *narya*, and *nenya*, are also expressed throughout the germarium with peak expression in regions 2A and 2B. Two recombination genes not captured in our analysis, *mei-W68* and *mei-P22*, share the same expression pattern as recombination genes that were found but are expressed in very few cells. Dot color represents average normalized expression level, and diameter represents fraction of cells expressing the gene in each cluster.

Finally, our strategy of focusing only on the top 500 genes failed to highly enrich for genes involved in recombination. Only 5 known recombination genes (*vilya*, *narya*, *nenya*, *Mcm5*, and *hdm*) made the top 500 and only one (*Mcm5*) had lines available with a phenotype in our assays. Many of the genes involved in DSB induction, such as the *spo-11* homolog, *mei-W68*, or meiotic DSB repair, are transcribed at much lower levels than SC genes (Fig. 10). Only a low level of transcript would be needed to produce enough protein to induce or repair approximately 2 dozen breaks. These transcripts may only be expressed in a subset of the cells expressing *vasa*, *c(3)G*, *cona*, and *corolla,* and their expression was not detectable in the larval ovary dataset that comprised part of the analysis. These factors would impact the strength of their co-expression ranking. The genes encoding the RING domain proteins *vilya*, *narya*, and *nenya* displayed higher expression levels, possibly due to these proteins playing multiple roles in recombination and therefore had expression patterns significant enough for the top 500 (Fig. 10). Using recombination genes as the markers for analyzing single-cell transcriptomics, data may be necessary to identify new genes that play roles in recombination. These results emphasize that when using single-cell transcriptiomics data to identify new genes, the marker genes for the analysis must be chosen carefully.

### Comparisons to other approaches to identify meiotic genes

Recently, other labs have used transcriptomics to try to identify new meiotic genes. In the study by the Nystul lab (Rust *et al*. 2020), the authors proposed 60 genes that could encode SC components based on expression patterns in their single-cell data. The study did not test any of the proposed genes for function in the germline. While half of the predicted genes were uncharacterized, only 5 of those uncharacterized genes fell within our top 500 list, and RNAi lines were only available for 3. Of those, only *CG4570* had a phenotype that indicated a role more in development than as an SC component. Of the 30 named genes identified as potential SC components in the Nystul lab analysis, only 7 were in our top 500 list. The line targeting *RPA3* caused the formation of underdeveloped ovaries, while the line targeting *enc* displayed SC maintenance and developmental defects most likely caused by the reported roles of Enc in dorsal-ventral patterning and oocyte differentiation (see Supplementary Table 3) (Hawkins *et al*. 1996; Van Buskirk *et al*. 2000). Another study using available RNAi lines focused on testing *CG* designated genes with reported germline expression in FlyAtlas by screening for chromosome nondisjunction and sterility (Sun *et al*. 2023). Since less than half of the *CG* genes on our list had available RNAi lines, the 2 analyses had only 3 genes identified as positives (*CG8142*, *CG10336*, and *CG6693*) in common.

Based on our results, genes required for early oocyte development may well outnumber those involved in meiotic chromosome dynamics on our list. The strategy of using co-expression patterns to identify new meiosis genes might be more fruitful by the addition of other parameters to prioritize candidates for testing. Recently, the Rog lab (Kursel *et al*. 2021) used protein structure and size to identify a new SC gene in a worm species.

In the future, a combination of using expression data followed by prioritizing candidates to analyze with structural homology might provide a high probability of finding rapidly evolving genes like meiotic genes. Identifying rapidly evolving genes requires using a variety of strategies. Indeed, our study demonstrates that using expression profiles and existing reagents can yield new information on the proteins involved at a specific stage of development. Our identification of 6 uncharacterized genes with roles in ovarian development and new phenotypes for additional genes indicates that single-cell transcriptomics combined with screening can be a useful tool for understanding gene function.

## Supplementary Material

jkae028_Supplementary_Data

## Data Availability

Original data underlying this manuscript can be accessed from the Stowers Original Data Repository at http://www.stowers.org/research/publications/libpb-1729. The Drosophila RNAi stocks utilized in this study were created by the Harvard Transgenic RNAi project (TRiP collection) (Zirin *et al*. 2020) and can be ordered from the Bloomington Drosophila Stock Center (BDSC). Supplemental material available at G3 online.
