# Supplementary material for: A transcriptomics-based RNAi screen for regulators of meiosis and early stages of oocyte development in Drosophila melanogaster
Source: G3 (Bethesda). 2024 Feb 9;14(4):jkae028. doi: 10.1093/g3journal/jkae028 (PMC10989863; doi:10.1093/g3journal/jkae028)
Supplement: jkae028_Supplementary_Data [file jkae028_supplementary_data.zip › Supplementary_Table_1_G3-2023-404773.docx]

**Supplementary Table 1.** **fastq files from NCBI Short Read Archive**

| SRA# | ID | nFeature_RNA cutoff | Percent.mt cutoff |
| --- | --- | --- | --- |
| SRX9588226 | Slaidina_1_adult | 775 < x < 6250 | < 5% |
| SRX9588227 | Slaidina_2_adult | 775 < x < 6250 | < 5% |
| SRX9588228 | Slaidina_3_adult | 775 < x < 6250 | < 5% |
| SRX9588229 | Slaidina_4_adult | 775 < x < 6250 | < 5% |
| SRX9588230 | Slaidina_5_adult | 775 < x < 6250 | < 5% |
| SRR9161641 | Slaidina _1_larval | 775 < x < 5500 | < 5% |
| SRR9161642 | Slaidina _2_larval | 775 < x < 5500 | < 5% |
| SRX8541186 | Rust_2_adult | 775 < x < 5500 | < 5% |
| SRX8541187 | Rust_3_adult | 775 < x < 5500 | < 5% |
