# Supplementary material for: A transcriptomics-based RNAi screen for regulators of meiosis and early stages of oocyte development in Drosophila melanogaster
Source: G3 (Bethesda). 2024 Feb 9;14(4):jkae028. doi: 10.1093/g3journal/jkae028 (PMC10989863; doi:10.1093/g3journal/jkae028)
Supplement: jkae028_Supplementary_Data [file jkae028_supplementary_data.zip › Supplementary_Table_2_G3-2023-404773.docx]

**Supplementary Table 2.** **Seurat analysis parameters**

| Analysis | # PC’s | Clustering Resolution |
| --- | --- | --- |
| Slaidina-adult | 100 | .5 |
| Slaidina-larval | 100 | .5 |
| Rust-adult | 100 | 1.25 |
| All | 50 | 0.8 |
